# Development of a core descriptor set for studies assessing interventions for diabetes-related foot ulceration

**DOI:** 10.1007/s00125-026-06815-1

**Published:** 2026-07-25

**Authors:** Aleksandra Staniszewska, Amy Jones, Luke Davies, Frances Game, Jane Nixon, David Russell, Sicco A. Bus, Jayer Chung, Vivienne Chuter, Ketan Dhatariya, Michael Edmonds, Robert Fitridge, Catherine Gooday, Emma J. Hamilton, Venu Kavarthapu, Joseph L. Mills, Matilde Monteiro-Soares, Edgar J. G. Peters, Joseph Shalhoub, Jaap J. van Netten, Dane K. Wukich, Robert J. Hinchliffe

**Affiliations:** 1 Translational Health Sciences, Bristol Medical School, University of Bristol, Bristol, UK; 2 North Bristol NHS Trust, Bristol, UK; 3 University Hospitals of Derby and Burton NHS Foundation Trust, Derby, UK; 4 Leeds Institute of Health Sciences, University of Leeds, Leeds, UK; 5 Leeds Institute of Clinical Trials Research, University of Leeds, Leeds, UK; 6 Amsterdam UMC, University of Amsterdam, Rehabilitation Medicine, Amsterdam Movement Sciences, Amsterdam, the Netherlands; 7 Division of Vascular Surgery and Endovascular Therapy, Michael E. DeBakey Department of Surgery, Baylor College of Medicine, Houston, TX USA; 8 School of Health Sciences, Western Sydney University, Campbelltown, NSW Australia; 9 Elsie Bertram Diabetes Centre, Norfolk and Norwich University Hospitals NHS Foundation Trust, Norwich, UK; 10 Norwich Medical School, University of East Anglia, Norwich, UK; 11 Diabetic Foot Clinic, King’s College Hospital NHS Foundation Trust, London, UK; 12 Discipline of Surgery, Adelaide University, Vascular and Endovascular Surgery Service, Royal Adelaide Hospital, Adelaide, SA Australia; 13 Norfolk and Norwich University Hospitals NHS Foundation Trust, Norwich, UK; 14 Medical School, University of Western Australia and Department of Endocrinology and Diabetes, Fiona Stanley Hospital, Murdoch, WA Australia; 15 Department of Trauma and Orthopaedics, King’s College Hospital, London, UK; 16 Portuguese Red Cross Health School Lisbon, Lisbon, Portugal; 17 Cross I&D: Research Center, Lisbon, Portugal; 18 CINTESIS @ RISE – Health, Faculty of Medicine, University of Porto, Porto, Portugal; 19 Amsterdam UMC location Vrije Universiteit Amsterdam, Infectious Diseases, Amsterdam, the Netherlands; 20 Amsterdam Movement Sciences, Rehabilitation and Development, Amsterdam, the Netherlands; 21 Amsterdam Institute for Immunology and Infectious Diseases, Infectious Diseases, Amsterdam, the Netherlands; 22 Imperial Vascular Unit, Imperial College Healthcare NHS Trust, London, UK and Department of Surgery & Cancer, Imperial College London, London, UK; 23 Department of Prosthetics and Orthotics, University of Texas Southwestern School of Health Professions, Dallas, TX USA

**Keywords:** Consensus, Delphi method, Foot ulceration, Methodology

## Abstract

**Aims/hypothesis:**

Foot ulceration is a common complication of diabetes and is associated with high mortality and costs. The quality of evidence to inform clinical practice is limited, partly because clinical studies do not consistently report baseline participant characteristics. This study aimed to develop a core descriptor set (CDS), a minimum set of descriptors to be measured in all studies evaluating interventions for people with diabetes-related foot ulceration.

**Methods:**

A longlist of descriptors was generated through a systematic review of studies assessing interventions for diabetes-related foot ulcers, pre-registered with PROSPERO (CRD42019128250). The identified descriptors were then ranked based on perceived importance by healthcare professionals from different fields and geographical locations using a nine-point Likert scale in the first round of a Delphi survey. Using standardised criteria, descriptors without consensus were re-ranked in round two. Critical descriptors and those without consensus after the Delphi process were discussed in the consensus meeting to finalise the CDS.

**Results:**

The systematic review yielded 95 candidate descriptors. The two Delphi rounds were completed by 102 and 69 healthcare professionals, respectively. The Delphi process identified 34 critically important descriptors and 13 descriptors without consensus, which were discussed in the consensus meeting. The ratified CDS included 28 descriptors across nine domains: demographic variables; individual factors; ulcer characteristics; limb characteristics; ongoing medical interventions; previous surgical interventions; medication history; biochemical measurements; and quality of life/function/symptoms.

**Conclusions/interpretation:**

This CDS reflects characteristics important to health professionals and researchers when reporting clinical studies on diabetes-related foot ulceration. Its use will aid the reporting of future studies.

**Graphical Abstract:**

**Figure:**
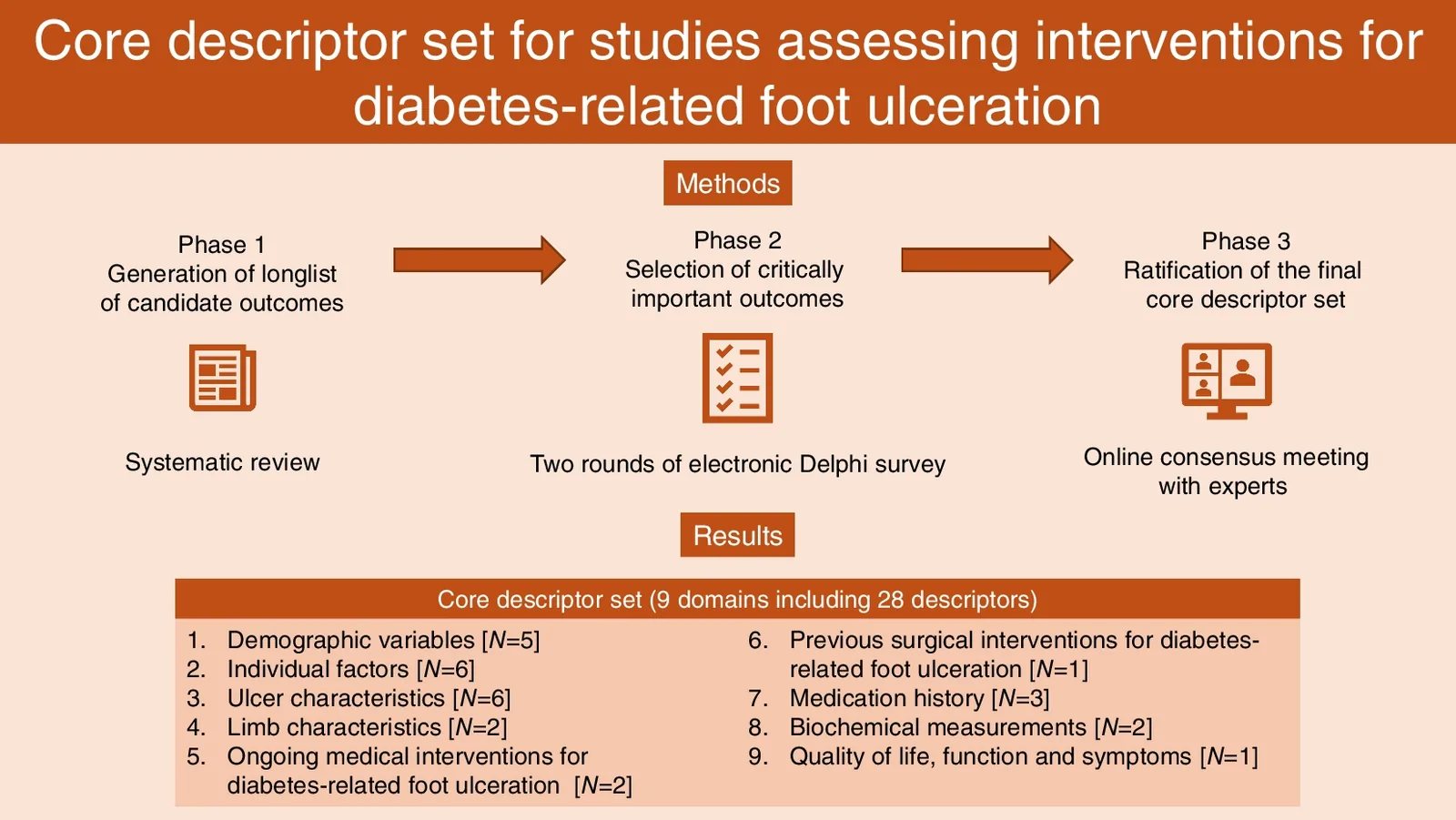

**Supplementary Information:**

The online version contains peer-reviewed but unedited supplementary material available at https://doi.org/10.1007/s00125-026-06815-1.

**Figure:**
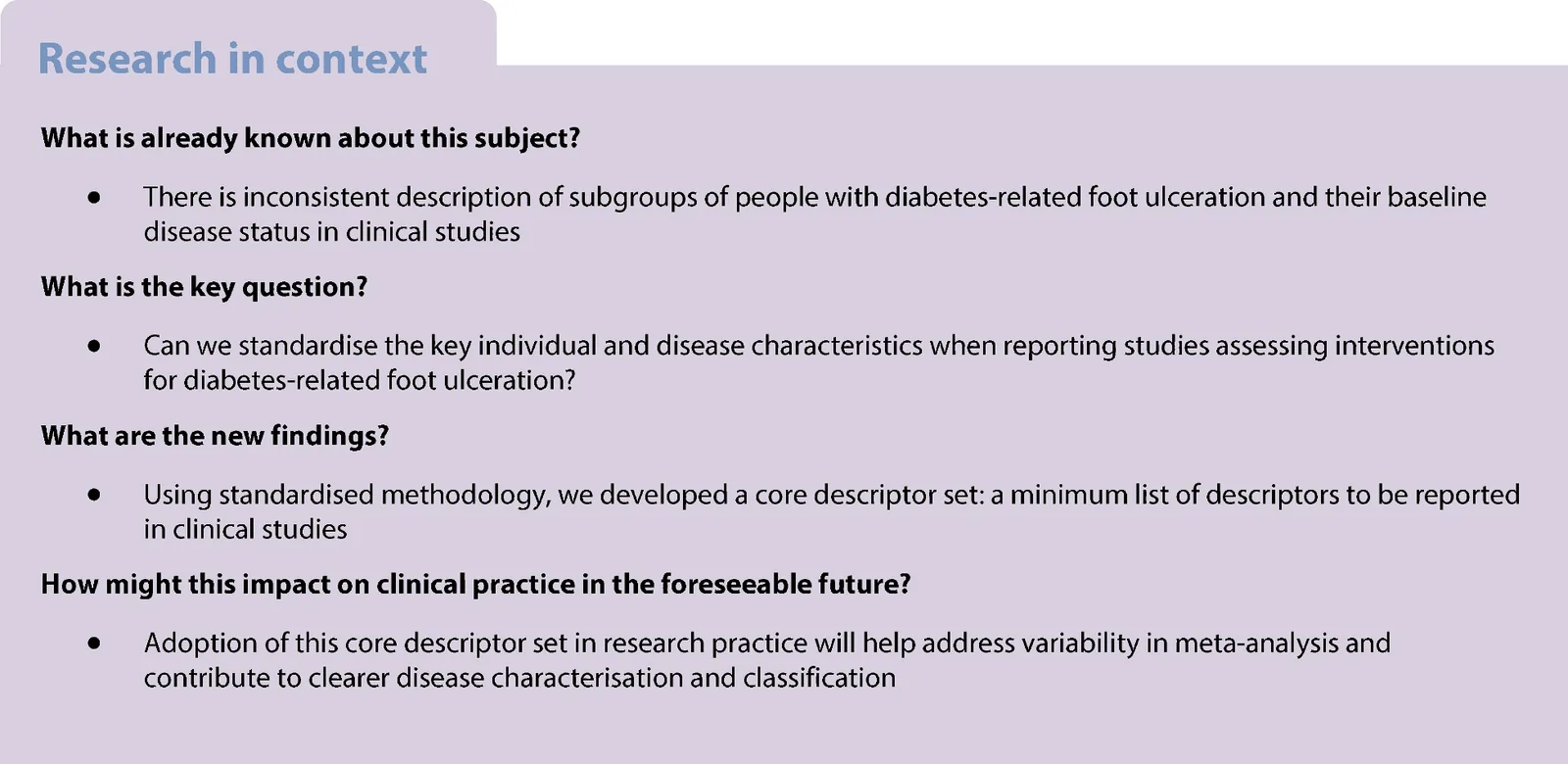

## Introduction

The burden of diabetes is increasing globally, with its estimated prevalence reaching 10% by 2050 [1]. Diabetes-related lower-limb complications, including foot ulceration, are a common problem, reducing quality of life and life expectancy [2, 3].

International evidence-based guidelines help inform management but the most effective treatments remain uncertain, in part because the quality of data underpinning clinical guidelines is relatively weak [4]. Efforts to improve the methodological quality of clinical studies in people with diabetes-related foot ulceration include the publication of a core outcome set, an agreed standardised set of outcomes that should be measured and reported, as a minimum, in all clinical studies in the field [5].

One of the major methodological challenges is the inconsistent description of subgroups of people with diabetes-related foot ulceration and their baseline disease status. Diabetes-related foot ulcers can differ significantly in aetiology, complexity, the need for additional medical interventions, and the likelihood of healing or progressing to amputation [6]. Incomplete or inconsistent reporting of baseline participant characteristics leads to erroneous conclusions about treatment effectiveness and limits the ability to synthesise studies [7].

It is essential to summarise participant and disease characteristics before any intervention is delivered in clinical studies. Recording these variables in a standardised fashion improves the internal validity (by allowing ascertainment of the comparability between groups under comparison) and external validity (by enabling the assessment of generalisability) of research findings [8]. Standardising the structure and content of the data to be collected at baseline promotes efficiency in trial design and data collection, eases the interpretation of between-study variability in meta-analysis and contributes to clearer disease characterisation and classification [9].

A core descriptor set (CDS) is a minimum set of agreed descriptors created in collaboration with stakeholders, including healthcare professionals and researchers, that should be measured and reported in all studies, in this case, evaluating interventions for diabetes-related foot ulceration [9]. The development of a CDS ensures that all studies report a basic set of descriptors allowing comparisons between different interventions, including pooling for systematic review, meta-analysis and meta-regression. The use of a CDS is good research practice and improves research quality, ultimately benefiting clinical care [9]. CDSs have been published in other areas, including perforated peptic ulcer disease, parastomal hernia and Crohn’s anal fistula, but not in diabetes-related foot disease [10–12].

The aim of this study was to develop a CDS for use in studies assessing treatments for people with diabetes-related foot ulceration.

## Methods

The study was designed and delivered in four phases using established methodology for development of a CDS [10–12] based on modified COMET guidelines [13]. While CDS development methodology differs from that of a core outcome set, the principles are similar, allowing the use of the Core Outcome Set – Standards for Reporting Statement (COS-STAR) to guide study reporting [14]. This study was approved by the University of Bristol Faculty of Health Sciences Research Ethics Committee (Ref.: 2024-18079-20352).

### Scope of the CDS

The CDS has been developed for use by all trials and clinical studies evaluating interventions for diabetes-related foot ulceration in adults aged ≥16 years with a diagnosis of diabetes.

### Steering group formation

The study was led by a multidisciplinary steering group with expertise in managing diabetes-related foot ulceration and delivering research in this field. The steering group, who have previously overseen the development of the core outcome set for studies assessing treatments for diabetes-related foot ulceration [5], consisted of three vascular surgeons (RJH, DR, AS), a diabetologist (FG), a trial methodologist (JN) and a public representative with lived experience of diabetes-related foot ulceration and experience in the delivery of Delphi-based studies. The role of the steering group was to facilitate the delivery of all four stages of the study.

### Phase I: generation of the longlist of candidate descriptors (August–September 2024)

The initial longlist of candidate descriptors was generated by reviewing publications that had been identified in a previously published systematic review of reported outcomes in people with diabetes-related foot ulceration [15]. The protocol for the systematic review was registered with PROSPERO (registration no. CRD42019128250). For this study, the reported participant and disease characteristic descriptors (rather than outcomes) were extracted by two independent reviewers (AJ and LD).

The identified descriptors were grouped by the steering group into ten domains, including demographic variables, individual factors, ulcer characteristics, limb characteristics, ongoing medical interventions, previous surgical interventions, medication history, biochemical measurements, physiological measurements and quality of life, function and symptoms.

The initial longlist of descriptors was subsequently reviewed by the steering group to exclude duplicates and rationalise the use of specific classification tools and tests.

### Phase II: Delphi process (September 2024–July 2025)

#### Delphi round 1 (September–December 2024)

The finalised longlist of candidate descriptors was entered into the first round of the electronic Delphi survey. The survey was delivered using the Research Electronic Data Capture (REDCap) platform hosted securely by the University of Bristol [16]. It consisted of a mandatory simplified electronic consent form, followed by questions related to the clinical expertise of the respondent. Survey participants were subsequently asked to rank candidate descriptors across ten domains using a nine-point Likert scale, with a score of 1–3 suggesting that the proposed descriptors were not important, a score of 4–6 corresponding to descriptors that were deemed to be important but not critical enough for inclusion in the core set, and a score of 7–9 highlighting critically important descriptors for consideration of inclusion in the final CDS.

An anonymous link to the survey was shared with healthcare professionals using mailing lists and newsletters of the key international societies supporting clinicians involved in the care of people with diabetes-related foot ulceration, including the International Working Group on the Diabetic Foot, Pan-Africa Diabetic Foot Study Group and the Vascular Society of Great Britain and Ireland’s Specialist Interest Group in the Diabetic Foot.

Given the heterogeneity of the population affected by diabetes-related foot ulceration, including significant variation in disease trajectory and underlying comorbidities, it was agreed that only healthcare professionals would be invited to participate in the Delphi process. This approach has been taken previously by research groups developing CDS in other disciplines [10–12] and was supported by the public representative in the steering group.

In line with previously used consensus criteria [5], descriptors rated 7–9 by ≥70% of participants were considered to be critically important and therefore automatically taken forward to the consensus meeting for consideration of inclusion in the final CDS. Conversely, descriptors rated 1–3 by ≥70% of respondents were regarded as unimportant and discarded. Descriptors not meeting the above criteria were deemed to lack consensus and were entered to round 2 for reprioritisation.

Delphi round 1 responses were screened for duplicates and if more than one survey was submitted from the same email address only the survey with the greatest proportion of descriptors ranked was included in the study.

#### Delphi round 2 (February–July 2025)

Following completion of the deduplication process, a personalised link to the second round of the Delphi survey was sent to study participants. For each descriptor, the respondents could see their previous response in relation to the median score of the whole group and a histogram showing the distribution of responses.

To improve the response rate, non-responders received up to three reminder emails from the REDCap platform.

The round 2 results were analysed using the consensus criteria adopted in the previous round.

### Phase III: steering group meeting and descriptor rationalisation (August 2025)

To ensure that the number of descriptors rated as either critical or without consensus after the two Delphi rounds could be discussed feasibly in the consensus meeting, the steering group rationalised the proposed list of items, ensuring that only those with the highest relevance to diabetes-related foot ulceration were taken forward for discussion.

### Phase IV: consensus meeting (September 2025)

The rationalised list of critical descriptors and those without consensus were subsequently discussed at the international multidisciplinary consensus meeting held online on the Microsoft Teams platform. The consensus meeting panel consisted of experts in diabetology, podiatry, infectious diseases, movement science, orthopaedic surgery and vascular surgery who have contributed to international diabetes-related foot ulceration diagnosis and management guidelines during their careers. The selected experts were identified purposefully from the members of the International Working Group on the Diabetic Foot and the Vascular Society of Great Britain and Ireland’s Specialist Interest Group in the Diabetic Foot.

The panel members rated the rationalised list of critical descriptors and those without consensus using interactive Mentimeter software (Menitmeter, Stockholm, Sweden) and discussed the rationale behind their choices with the rest of the group. The final CDS was ratified at the end of the consensus meeting. All consensus meeting participants signed written consent forms before participating.

## Results

**Fig. 1:**
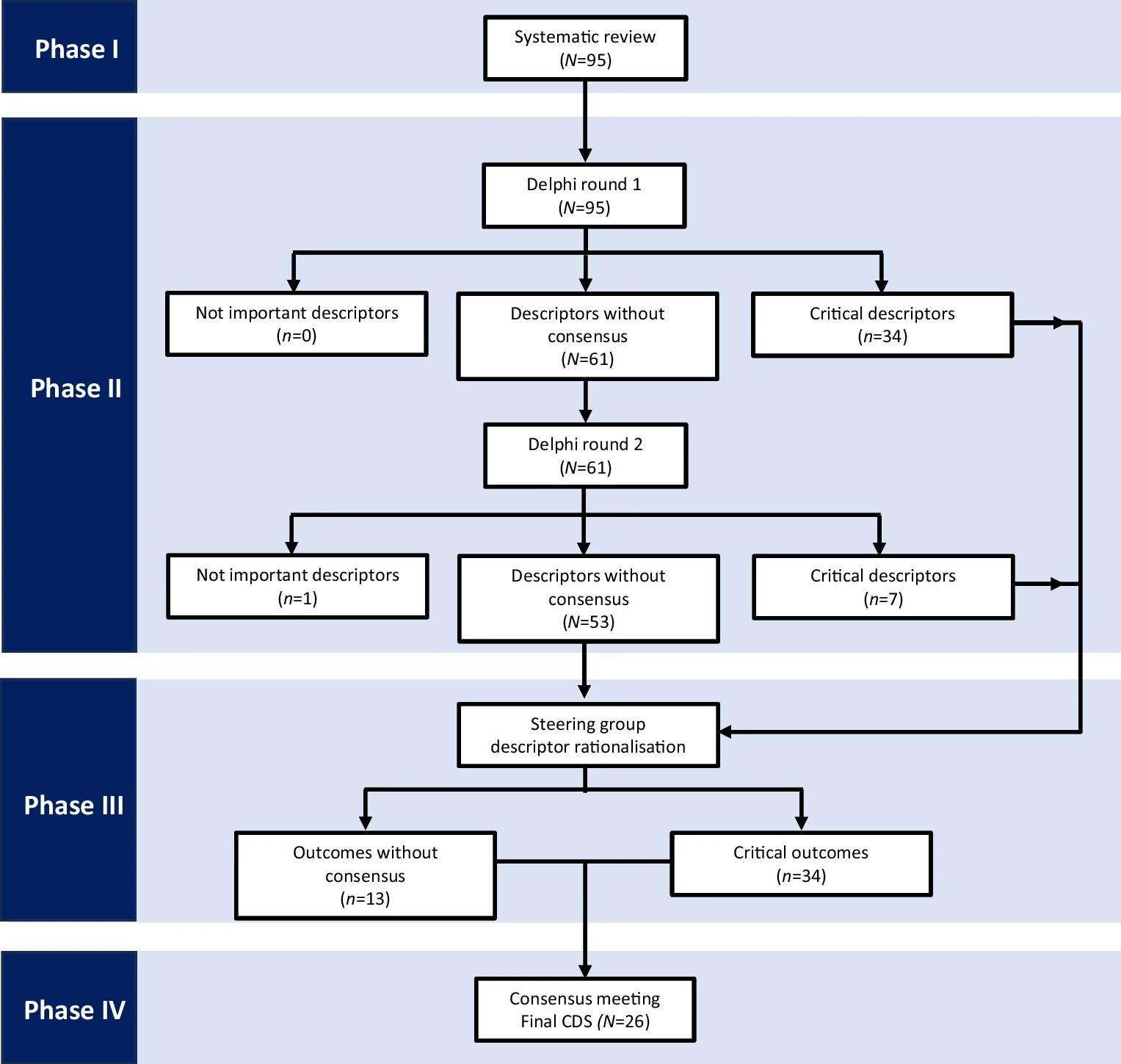
Flowchart demonstrating the results of each of the four phases of CDS development

The results of the four phases of CDS development are summarised in Fig. 1.

### Phase I: generation of the longlist of candidate descriptors (August–September 2024)

The systematic review of studies assessing the effectiveness of interventions for diabetes-related ulceration yielded 145 candidate descriptors, which were split into ten domains (electronic supplementary material [ESM] Table 1). It has been recognised that the Delphi response rate is inversely proportional to the number of items included [17]. To ensure that the number of descriptors being entered into round 1 of the Delphi survey was practical and not overwhelming to the respondents, the steering group reviewed the initial longlist of descriptors.

Following multidisciplinary discussion and input from our public representative, 32 items relating to individual blood tests were redistributed across seven new descriptors capturing groups of blood tests, including glycaemic management, renal function, lipid profile, liver function, measures of inflammation or infection, measures of metabolism and measures of coagulation. The 11 identified scoring systems, such as Site, Ischaemia, Neuropathy, Bacterial Infection, Area, and Depth system (SINBAD), Saint Elian Wound Score System (SEWSS) and Wound, Ischemia, and foot Infection classification (WIfI) [18], were split into their individual components, most of which had already been included in the longlist. This yielded six additional descriptors, including perfusion, number of ulcers, extent of the wound, extent of skin undermining, description of wound edges and ulcer depth. The steering group noted that 18 items were already captured within other descriptors (e.g. calcium channel blockers were represented within antihypertensives), two descriptors were merged into one, and ‘duration of healing’ was felt to be too difficult to define and capture in clinical practice and was therefore removed from the longlist. Following the first rationalisation exercise, the finalised longlist of descriptors taken forward to the first Delphi round included 95 descriptors across ten domains (ESM Table 1).

### Phase II: Delphi process (September 2024–July 2025)

The 95 unique candidate descriptors were entered into the first round of the Delphi survey. The two consecutive Delphi rounds were completed by 102 and 69 healthcare professionals between September 2024 and July 2025, resulting in 68% retention rate in the second round. Participating healthcare professionals represented 36 countries across six continents and were mainly doctors and podiatrists, with more than half having at least 20 years of clinical experience (Table 1).

**Table 1:** Demographics of healthcare professionals participating in the Delphi survey

Demographic	Round 1 (*N*=102)	Round 2 (*N*=69)
Location		
Europe	64 (63)	49 (71)
North America	10 (10)	9 (13)
South America	3 (3)	0 (0)
Africa	12 (12)	3 (4)
Australia	5 (5)	4 (6)
Asia	8 (8)	4 (6)
Clinical role of healthcare professionals		
Doctor	59 (58)	41 (59)
Podiatrist	22 (22)	18 (26)
Nurse	11 (11)	2 (3)
Other	10 (10)	8 (12)
Specialty of healthcare professionals		
Vascular surgery	13 (13)	11 (16)
Orthopaedics	2 (2)	2 (3)
Diabetes and endocrinology	24 (24)	15 (22)
Other	63 (62)	41 (59)
Seniority of participating doctors		
Consultant/attending	50 (85)	35 (85)
Staff grade	4 (7)	2 (5)
Registrar/resident	5 (8)	4 (10)
Length of clinical experience		
<5 years	6 (6)	3 (4)
5–10 years	14 (14)	10 (15)
11–20 years	27 (26)	16 (23)
>20 years	55 (54)	40 (58)

Values are provided as *n* (%)

Out of 95 candidate descriptors at the start of round 1, 34 (36%) were deemed to be critically important and were listed for consideration in the final CDS. The remaining 61 (64%) descriptors did not reach consensus and were taken forward to the second and final Delphi round. None of the descriptors was found to be unimportant in round 1.

Out of the 61 descriptors that entered round 2 of the Delphi process, 53 (87%) continued to lack consensus, seven (11%) were reprioritised as critical, and one (2%) was found to be unimportant and therefore excluded from further discussion. The results of the two Delphi rounds are summarised in Table 2.

**Table 2:** Results of the two rounds of the Delphi survey

Descriptor	Votes for exclusion (rated 1–3)		Votes for inclusion (rated 7–9)		Result
	Round 1	Round 2^a^	Round 1	Round 2^a^	
Domain I: demographic variables					
Age	1		78		Critical
Sex	8	6	58	74	Critical
BMI	33	3	67	71	Critical
History of smoking	1		80		Critical
Race/ethnicity	12	6	46	47	No consensus
Alcohol intake	12	15	36	18	No consensus
Employment/profession	16	9	26	14	No consensus
Education	11	14	35	23	No consensus
Marital status	36	38	15	6	No consensus
Urban status	21	25	23	11	No consensus
Neighbourhood income quintile	22	16	22	8	No consensus
Income	20	22	27	11	No consensus
Dietary habits	20	14	42	28	No consensus
Religion	63	77	9	6	Excluded
Family history of diabetes	28	28	34	22	No consensus
Domain II: individual factors					
Duration of diabetes	3		87		Critical
Heart disease	1		80		Critical
Hypertension	5	5	53	67	No consensus
Type of diabetes mellitus	6		70		Critical
Kidney disease	0		82		Critical
Hyperlipidaemia	5	3	59	59	No consensus
Peripheral arterial disease	0		100		Critical
Diabetes-related neuropathy	1		94		Critical
Diabetes-related retinopathy	3	6	67	68	No consensus
Prior diabetes-related foot ulcer	0		98		Critical
Prior stroke	2	5	56	66	No consensus
Diabetes-related nephropathy	1		83		Critical
Dialysis	0		90		Critical
Chronic pulmonary disease	19	13	22	17	No consensus
Central nervous system disorder	17	16	39	39	No consensus
Ophthalmic disorder	19	16	39	30	No consensus
Depression	10	6	61	64	No consensus
Anaemia	8	5	48	40	No consensus
Gastrointestinal disorder	34	31	19	9	No consensus
Arthritis	26	25	30	14	No consensus
Musculoskeletal disorder	17	16	40	27	No consensus
Genitourinary disorder	39	44	16	3	No consensus
Metabolic disorder	18	13	38	28	No consensus
Tumours	18	17	32	23	No consensus
Previous deep venous thrombosis	16	14	39	27	No consensus
Hepatobiliary disorder	35	30	21	6	No consensus
Domain III: ulcer characteristics					
Baseline ulcer size	1		94		Critical
Duration of ulceration	5		95		Critical
Ulcer location	1		97		Critical
Ulcer depth	2		91		Critical
Presence of wound infection	0		97		Critical
Extent of the wound	2		83		Critical
Type of bacterial wound infection	1		82		Critical
Cause of ulceration	3		72		Critical
Osteomyelitis	0		99		Critical
Number of ulcers	1		80		Critical
Peri-wound oedema	6	5	68	77	Critical
Ulcer symptoms	9	5	64	83	Critical
Extent of skin undermining	6		74		Critical
Description of wound edges	8	3	67	81	Critical
Domain IV: limb characteristics					
Perfusion	2		92		Critical
Foot pain	5		71		Critical
Laterality of the limb involved	17	11	52	56	No consensus
Vessel calcification	7	2	59	72	Critical
Skin temperature	10	13	55	50	No consensus
Postural instability	14	14	44	30	No consensus
Electromyography	28	38	20	2	No consensus
Domain V: ongoing medical interventions for foot ulceration					
Current use of antibiotics	0		82		Critical
Offloading treatments	0		99		Critical
Duration of antibiotic therapy	1		75		Critical
Number of dressing changes required	8	5	59	61	No consensus
Domain VI: previous surgical interventions for foot ulceration					
Previous amputation	0		95		Critical
Previous revascularisation	0		90		Critical
Domain VII: medication history					
Insulin therapy	4	2	58	55	No consensus
Oral glucose-lowering medications	6	3	53	55	No consensus
Statins	3	3	57	62	No consensus
Antiplatelets	6	5	57	63	No consensus
Antihypertensives	8	6	50	53	No consensus
Anticoagulants	6	3	52	64	No consensus
Domain VIII: biochemical measurements					
Glycaemic management	1		84		Critical
Renal function	2		84		Critical
Lipid profile	11	8	42	37	No consensus
Measures of inflammation or infection	2		81		Critical
Liver function	14	10	42	24	No consensus
Haemoglobin levels	5	3	65	60	No consensus
Measures of metabolism	24	28	23	9	No consensus
Coagulation	14	10	43	32	No consensus
Magnesium levels	30	34	17	8	No consensus
Domain IX: physiological measurements					
Systolic BP	13	10	44	38	No consensus
Heart rate	15	11	38	25	No consensus
Domain X: quality of life, functional status and symptoms					
Ambulatory status	3		80		Critical
Level of independence with the activities of daily living	1		70		Critical
Duration of physical activity per day	2	2	53	60	No consensus
Assessment of the level of disability caused by neuropathy	7	2	53	64	No consensus
Emotional burden	6	3	43	46	No consensus
Health-related quality of life	3	0	69	65	No consensus
Disease-specific quality of life	3	22	66	78	Critical
Assessment of illness perception	7	3	53	65	No consensus
Living arrangements	11	14	42	32	No consensus
Primary caregiver role	14	10	47	29	No consensus

Values are presented as the percentage of respondents selecting a specific rating for each descriptor

Critical descriptors for potential inclusion in CDS defined as ≥70% of respondents scoring as 7–9 (critical) and 1–3 by ≤15%; descriptors for exclusion defined as ≥70% of respondents scoring as 1–3 (not important) and 7–9 by ≤15%; indeterminate votes (neither for inclusion nor exclusion) are not presented in the table

^a^Only descriptors without consensus were rescored in the second round

### Phase III: steering group meeting and descriptor rationalisation (August 2025)

After the two Delphi rounds, 41 (43%) and 53 (56%) descriptors were rated as either ‘critically important’ or ‘without consensus’, respectively. Given the high potential number of descriptors requiring in-depth discussion in the consensus meeting, the steering group scrutinised and rationalised the list of items prior to the consensus meeting. Of the 41 items rated as critical in Delphi process, seven were excluded as three were already captured within other descriptors, three were considered to be too subjective and would not be reliably reported in the studies, and one was felt not to be as important as another very similar descriptor. Details of these decisions are provided in ESM Table 2.

Out of 53 descriptors not reaching consensus after the Delphi process, 40 were excluded as most lacked direct clinical relevance to diabetes-related foot ulceration, were not relevant to all healthcare settings or were captured in other descriptors (ESM Table 3).

Following the rationalisation process, 34 critical descriptors and 13 descriptors without consensus were brought forward for discussion in the consensus meeting.

### Phase IV: consensus meeting (September 2025)

The consensus meeting was attended by 18 international experts involved in the management of diabetes-related foot ulceration, including seven vascular surgeons, two orthopaedic surgeons, three podiatrists, three diabetologists, two movement scientists and one infectious diseases physician representing USA, UK, the Netherlands, Portugal and Australia.

Following a round of voting and interdisciplinary discussion as summarised in Table 3, the panel unanimously ratified the CDS for studies evaluating interventions for diabetes-related foot ulceration to include 28 descriptors across nine domains:

- Domain I (demographic variables), including age, sex, race and ethnicity, BMI and history of smoking
- Domain II (individual factors), including prior diabetes-related foot ulcer, diabetes-related neuropathy, duration of diabetes mellitus, type of diabetes, heart disease and chronic kidney disease stage
- Domain III (ulcer characteristics), including osteomyelitis, ulcer location, soft tissue infection/cellulitis, duration of ulceration, baseline ulcer size and ulcer depth
- Domain IV (limb characteristics), including perfusion and peripheral arterial disease
- Domain V (ongoing medical interventions for diabetes-related foot ulceration), including current use of antibiotics and offloading treatments
- Domain VI (previous surgical interventions for diabetes-related foot ulceration), including previous minor and major amputation
- Domain VII (medication history), including insulin therapy, oral glucose-lowering medications and non-insulin injectable agents
- Domain VIII (biochemical measurements), including glycaemic management and eGFR
- Domain IX (quality of life, function and symptoms), including health-related quality of life

**Table 3:** Results of the consensus meeting compared with Delphi survey scores

Descriptor	Participants voting for exclusion (rated 1–3)		Participants voting as no consensus (rated 4–6)		Participants voting for inclusion (rated 7–9)	
	Delphi	Consensus meeting	Delphi	Consensus meeting	Delphi	Consensus meeting
Voted as critical in the Delphi process						
Domain I: demographic variables						
Age	1	7	21	7	78	86
Sex	6	0	20	23	74	77
History of smoking	1	0	19	38	80	62
Domain II: individual factors						
Duration of diabetes mellitus	3	0	10	29	87	71
Heart disease	1	0	19	33	80	67
Type of diabetes mellitus	6	0	24	7	70	93
Peripheral arterial disease	0	0	0	0	100	100
Diabetes-related neuropathy	1	0	5	0	94	100
Dialysis	0	0	10	13	90	87
Prior diabetes-related foot ulcer	0	0	2	27	98	73
Domain III: ulcer characteristics						
Baseline ulcer size	1	0	5	7	94	93
Duration of ulceration	5	0	0	27	95	73
Ulcer location	1	0	2	7	97	93
Ulcer depth	2	0	7	13	91	87
Presence of wound infection	0	0	3	7	97	93
Extent of the wound	2	0	15	79	83	21
Osteomyelitis	0	0	1	13	99	87
No. of ulcers	1	0	19	40	80	60
Domain IV: limb characteristics						
Perfusion	2	0	6	27	92	73
Foot pain	5	20	24	67	71	13
Domain V: ongoing medical interventions for foot ulceration						
Current use of antibiotics	0	7	18	36	82	57
Offloading treatments	0	0	1	7	99	93
Duration of antibiotic therapy	1	7	24	73	75	20
Domain VI: previous surgical interventions for foot ulceration						
Previous amputation	0	0	5	7	95	93
Previous revascularisation	0	0	10	29	90	71
Domain VIII: biochemical measurements						
Glycaemic management	1	0	15	14	84	86
Renal function	2	7	14	27	84	67
Measures of inflammation/infection	2	14	17	71	81	14
Domain X: quality of life, functional status and symptoms						
Ambulatory status	3	0	17	19	80	81
The level of independence with the activities of daily living	1	13	29	31	70	56
Disease-specific quality of life	22	0	0	38	78	63
No consensus following the Delphi process						
Domain I: demographic variables						
Race and ethnicity	6	14	47	43	47	43
Domain II: individual factors						
Hypertension	5	14	28	86	67	0
Diabetes-related retinopathy	6	25	26	67	68	8
Prior stroke	5	29	29	64	66	7
Chronic pulmonary disease	13	20	70	67	17	7
Depression	6	0	30	93	64	7
Anaemia	5	29	55	64	40	7
Domain V: ongoing medical interventions for foot ulceration						
No. of dressing changes required	5	40	44	60	61	0
Domain VII: medication history						
Insulin therapy	2	0	43	60	55	40
Oral glucose-lowering medications	3	20	42	47	55	33
Statins	3	7	35	60	62	33
Antiplatelet agents	5	0	32	73	63	27
Domain X: quality of life, functional status and symptoms						
Health-related quality of life	0	0	35	0	65	100

Values are presented as percentage of respondents selecting a specific rating for each descriptor

Descriptors for potential inclusion in CDS were defined as ≥70% of respondents scoring as 7–9 (critical) and 1–3 by ≤15%; descriptors for exclusion were defined as ≥70% of respondents scoring as 1–3 (not important) and 7–9 by ≤15%; descriptors with consensus were defined as not meeting criteria for inclusion or exclusion

## Discussion

### Key findings

This CDS is a result of a large interdisciplinary and international effort to standardise the key participant and disease characteristics when reporting studies assessing interventions for diabetes-related foot ulceration. This CDS is the minimum list of descriptors to be reported but does not provide specific guidance on how these descriptors should be measured, nor prohibits individual studies from including additional descriptors based on their individual design.

### CDS in the context of previous work

This CDS was created using the internationally recognised and validated COMET framework [13] that has guided development of CDS in other disciplines [10–12]. The development process has been reported in line with COS-STAR guidance [14]. The International Working Group on the Diabetic Foot has previously published recommendations on what participant and disease characteristics ought to be described and what outcomes should be included when reporting studies addressing management of diabetes-related foot ulceration [19]. These recommendations, however, were an expert opinion rather than a formal study using a defined methodology. Nonetheless, 15 out of 17 recommended descriptors included in that opinion letter featured in our CDS, with the two exceptions being the number of ulcers and presence of foot deformity. This suggests overall alignment of this CDS with the expert international opinion. Our group has previously published a core outcome set for studies assessing interventions for diabetes-related foot ulceration [5] to standardise outcome reporting across trials. The addition of this CDS further strengthens our international efforts to improve the overall quality of the evidence underpinning clinical practice by allowing comparison of studies evaluating similar populations.

### Ratification of the final CDS

The consensus meeting allowed for in-depth interdisciplinary discussion among experts, bringing a range of perspectives and clinical backgrounds. The panel aimed to ratify a balanced CDS that included a wide range of descriptors to encourage comprehensive characterisation of the study population while remaining useable and practicable. The final ratified CDS includes 28 descriptors spanning nine domains, which is comparable with sets in other disciplines including between 19–37 descriptors across six to eight domains [10–12].

### Participant characteristics

Our final CDS includes basic demographic characteristics, such as sex, age, race and ethnicity, the reporting of which has been required by leading research funders globally to help achieve health equity. The NIH Revitalisation Act of 1993, with subsequent amendments in 2000 and 2007, stipulated that female sex and members of the minority groups should be included in clinical research [20]. Since 2017, the world leading clinical trials database, ClinicalTrials.gov, has additionally mandated reporting of race and ethnicity for all registered studies on their platform [21]. Furthermore, since 2025, the NIHR in the UK has mandated demonstration of equal sex inclusion throughout research lifecycle [22]. To tackle possible age discrimination in research, 42 main research funders and charities in the UK have released a joint statement to promote inclusion of older adults in clinical research [23].

The two additional descriptors included in this domain were BMI and history of smoking. Previous studies demonstrated J-shaped association between BMI and risk of diabetes-related foot ulceration [24] and underweight individuals with established foot ulcers have been found to have higher mortality and increased risk of limb loss [25]. Cigarette smoking has been linked to increased oxidative stress worsening diabetes-related neuropathy and impairing all four phases of wound healing [26] as well as higher major amputation risk [25].

### Individual factors

The key individual factors included in our CDS have been linked to high risk of ulcer recurrence and were found to be important predictors of all-cause mortality [27–30]. Inclusion of history of prior diabetes-related foot ulcer was essential as approximately 40% of ulcers recur within 1 year of healing [30] and those with recurrent ulcers were found to have had a minor amputation, and to have a longer duration of diabetes and diabetes-related neuropathy [27, 28]. Time to ulcer recurrence was found to be longer in individuals with no prior history of ulceration [29].

The optimal reporting of renal disease as a comorbidity generated significant discussion during the consensus meeting. The Delphi survey contained four separate descriptors referring to renal disease, including diabetes-related nephropathy, dialysis, kidney disease and renal function. On balance, the consensus meeting group felt that the burden of renal disease would be most effectively captured as chronic kidney disease stage. This approach is in keeping with how renal disease has been reported in clinical trials over the past two decades [31].

#### Ulcer and limb characteristics

Given the multitude of classification and scoring systems for diabetes-related foot ulceration, with only a minority being developed in large multicentre studies, having undergone external validation and assessment of reliability, none were included in this CDS. Instead, individual descriptors relating to ulcer and limb characteristics were identified through the Delphi process and subsequently ratified in the consensus meeting. The selected descriptors could be, however, retrospectively translated into the key classification systems recommended by the International Working Group on the Diabetic Foot: SINBAD system, Infectious Diseases Society of America/IWGDF (IDSA/IWGDF) classification and the WIfI system [18].

#### Previous or ongoing medical interventions for diabetes-related foot ulceration

Our CDS includes current use of antibiotics, as antimicrobial therapy has a synergistic effect when delivered in conjunction with other interventions and is recommended following surgical debridement of severe soft tissue infections [32]. Prompt treatment of infection in the context of diabetes-related ulceration is essential in reducing the risk of sepsis and limb loss [33]. Despite this, the Concordance in Diabetic Foot Ulcer Infection 2 (CODIFI2) study showed that 16.1% of individuals with suspected infection are not on any antimicrobial therapy [34].

Furthermore, we recommend reporting of any current use of offloading modalities when characterising the study population, as offloading therapy is frequently delivered together with other interventions [35].

#### Previous surgical interventions for diabetes-related foot ulceration

While many surgical interventions for diabetes-related foot ulceration exist, our CDS only incorporates amputation. This descriptor, however, includes both minor (either ipsilateral or contralateral) and contralateral major amputation. History of minor amputation is a predictor of ulcer recurrence [27, 28], and previous contralateral major amputation affects suitability for various offloading modalities [36] and is linked to 70% of the 5 year mortality rate [30]. History of revascularisation was not included in the final set, as it was felt that it would not necessarily reflect current perfusion status, which is already captured by descriptors in the limb characteristics domain.

#### Medication history

In addition to the inclusion of standard glucose-lowering agents such as insulin and oral glucose-lowering medications, the consensus group recommended the inclusion of novel non-insulin injectable agents. While these were not identified through our systematic review, an increasing number of studies have recently addressed the impact of novel injectables on diabetes-related foot ulcer healing [37].

#### Biochemical measurements

The two biochemical measurements included in the CDS are glycaemic management and eGFR. Understanding the effectiveness of glycaemic management in the studied population is essential when evaluating wound healing, as chronic hyperglycaemia may promote persistent inflammation in diabetes-related foot ulcers through modulation of numerous metabolic pathways [38]. eGFR was felt to be the most universally used measure of renal function [39] that is likely to be recorded routinely for individuals with diabetes-related foot ulcers. It has been incorporated into the CDS alongside chronic kidney disease stage as it was argued that chronic kidney disease may be present despite normal eGFR [39]. Thus, these two measures were included to effectively capture the burden of renal disease in most clinical studies.

#### Quality of life, function and symptoms

Health-related quality of life was ratified as the most informative descriptor to capture the level of independence with activities of daily living and emotional burden of the disease. Furthermore, as it is featured in our previously published core outcome set for studies evaluating the interventions for diabetes-related foot ulceration [5], direct impact of the intervention on the health-related quality of life could be measured and economic evaluations undertaken.

### Strengths and limitations

Our CDS is a result of an international effort involving clinicians and researchers from 36 countries across six continents using recognised COMET methodology [10–13]. The study participants represent a wide spectrum of clinical disciplines, including podiatry, diabetology, and vascular and orthopaedic surgery, providing a range of perspectives. Our multidisciplinary expert consensus group involved in the ratification of the final CDS was composed of leaders in the field of diabetes-related foot disease who are heavily involved in the development of international guidelines on prevention, classification and management of diabetes-related foot ulcers. Furthermore, we involved a person living with diabetes throughout the lifecycle of our study. With their help, we were able to ensure that descriptors that may be important to them were not eliminated during the descriptor rationalisation stages of the study. Additionally, they provided insight into how certain descriptors may be perceived, prompting us to ensure that even if not included in the final CDS, they would be captured within similar descriptors (e.g., the level of independence with the activities of daily living could be captured in health-related quality of life).

Despite strong methodology, our study is not without limitations. Even with our best efforts to maximise the response rate in the second survey round, it was ultimately only completed by 68% of study participants. This may represent overall fatigue with respect to survey-based studies and lack of time due to competing commitments. Nonetheless, the observed response rate is comparable with that in other Delphi studies [5, 10, 12]. Similar to our previous experiences [5] and those of other groups [40], study participants struggled to be highly selective when rating individual descriptors during the Delphi stage of the project. As a result, 41 (43%) of descriptors were rated as critical and were highlighted for consideration in the CDS. Only one descriptor was found to be unimportant and therefore excluded, and after two rounds of Delphi, the remaining 53 (56%) descriptors did not reach consensus and required further discussion. To ensure that the final CDS was practicable and relevant in research practice, the steering group had to rationalise the list of candidate descriptors prior to the consensus meeting to allow for a thoughtful and in-depth discussion of the final contents of the CDS. This approach has been successfully used before by our group during the development of a core outcome set for interventions for diabetes-related foot ulceration [5].

### Impact and implementation strategy

This CDS was developed to standardise characterisation of participants when reporting clinical studies evaluating treatments for diabetes-related foot ulceration. It is designed to be used in conjunction with our previously developed core outcome set, which defined a minimum list of outcomes that should be measured when evaluating interventions for diabetes-related foot ulcers [5].

This work will be shared with key international societies and interest groups fostering research in diabetes-related foot ulceration. Adoption of this CDS in research practice will help tackle variability in meta-analysis and contribute to clearer disease characterisation and classification.

## Supplementary Information

Below is the link to the electronic supplementary material.

ESM Tables (PDF 237 KB)

## Data Availability

All data generated or analysed during this study are included in this published article and its ESM.

## Abbreviations

CDS: Core descriptor set
COS-STAR: Core Outcome Set – Standards for Reporting Statement
REDCap: Research Electronic Data Capture
SEWSS: Saint Elian Wound Score System
SINBAD: Site, Ischaemia, Neuropathy, Bacterial Infection, Area, and Depth
WIfI: Wound, Ischemia, and foot Infection
