# Supplementary material for: Development of a core descriptor set for studies assessing interventions for diabetes-related foot ulceration
Source: Diabetologia. 2026 Jul 25;69(10):2784–97. doi: 10.1007/s00125-026-06815-1 (PMC13541978; doi:10.1007/s00125-026-06815-1)
Supplement: Supplementary file 1 [file 125_2026_6815_MOESM1_ESM.pdf]

Descriptor domain	Descriptor as identified through systematic review	Number of studies reporting	Comments	Descriptor as taken through to Delphi Survey round 1 after rationalisation by the steering group	Number of studies reporting	Comments
**Demographic variables**	Age	101		Age	101	
	Sex	100		Sex	100	
	Body mass index (BMI)	47		BMI	47	
	History of smoking	40		History of smoking	40	
	Race/ethnicity	17		Race/ethnicity	17	
	Alcohol intake	11		Alcohol intake	11	
	Employment/profession	4		Employment/profession	4	
	Education	3		Education	3	
	Marital status	3		Marital status	3	
	Urban status	2		Urban status	2	
	Neighbourhood income quintile	1		Neighbourhood income quintile	1	
	Income	1		Income	1	
	Dietary habits	1		Dietary habits	1	
	Religion	1		Religion	1	
	Family history of diabetes mellitus	1		Family history of diabetes mellitus	1	
**Individual factors**	Duration of diabetes mellitus	44		Duration of diabetes mellitus	44	
	Heart disease	30		Heart disease	30	
	Hypertension	28		Hypertension	28	
	Type of diabetes mellitus	27		Type of diabetes mellitus	27	
	Kidney disease	19		Kidney disease	19	
	Hyperlipidaemia	17		Hyperlipidaemia	17	
	Peripheral arterial disease	15		Peripheral arterial disease	15	
	Diabetes-related neuropathy	13		Diabetes-related neuropathy	13	
	Diabetes-related retinopathy	10		Diabetes-related retinopathy	10	
	Prior stroke	8		Prior stroke	8	
	Diabetes-related nephropathy	7		Diabetes-related nephropathy	7	
	Dialysis	6		Dialysis	6	
	Chronic pulmonary disease	5		Chronic pulmonary disease	5	
	Depression	2		Depression	2	
	Ophthalmic disorder	2		Ophthalmic disorder	2	
	Central nervous system disorder	2		Central nervous system disorder	2	
	Anaemia	2		Anaemia	2	
	Prior diabetes-related foot ulcer	1		Prior diabetes-related foot ulcer	10	
	Gastrointestinal disorder	1		Gastrointestinal disorder	1	

Descriptor domain	Descriptor as identified through systematic review	Number of studies reporting	Comments	Descriptor as taken through to Delphi Survey round 1 after rationalisation by the steering group	Number of studies reporting	Comments
**Individual factors (continued)**	Arthritis	1		Arthritis	1	
	Musculoskeletal disorder	1		Musculoskeletal disorder	1	
	Genitourinary disorder	1		Genitourinary disorder	1	
	Metabolic disorder	1		Metabolic disorder	1	
	Tumours	1		Tumours	1	
	Previous deep vein thrombosis	1		Previous deep vein thrombosis	1	
	Hepatobiliary disorder	1		Hepatobiliary disorder	1	
**Ulcer characteristics**	Baseline ulcer size	66		Baseline ulcer size	66	
	Duration of disease	47		Duration of ulceration	47	
	Ulcer location	46		Ulcer location	46	
	Wagner ulcer type	23	Captured in other descriptors (depth, infection, osteomyelitis)	Ulcer depth	26	Component of a scoring system listed in limb characteristics
	Presence of wound infection	19		Presence of wound infection	19	
	History of ulcer	9	Captured in prior diabetes-related ulcer in patient co-morbidities domain	Extent of the wound	18	Component of a scoring system listed in limb characteristics
	Type of bacterial infection	7		Type of bacterial wound infection	7	
	Cause of ulceration	6		Cause of ulceration	6	
	Osteomyelitis	5		Osteomyelitis	5	
	Ischemic ulcer	5	Captured in perfusion/peripheral arterial disease	Number of ulcers	4	Component of a scoring system listed in limb characteristics
	Number of ulcers	4		Peri-wound oedema	3	
	Laterality of limb involved	3	Moved to limb characteristics domain	Ulcer symptoms	3	
	Peri-wound oedema	3		Extent of skin undermining	1	Component of a scoring system listed in limb characteristics
	Ulcer symptoms	3		Description of wound edges	1	Component of a scoring system listed in limb characteristics
**Limb characteristics**	Ankle-brachial pressure index (ABPI)	23	Captured in perfusion	Perfusion	52	Component of a scoring system listed in limb characteristics
	Texas University Classification (TUC)	11	Split into individual components	Foot pain	9	
	Foot pain	9		Laterality of the limb involved	3	
	WIfI classification	5	Split into individual components (perfusion, infection, extent of the wound)	Vessel calcification	1	

Descriptor domain	Descriptor as identified through systematic review	Number of studies reporting	Comments	Descriptor as taken through to Delphi Survey round 1 after rationalisation by the steering group	Number of studies reporting	Comments
**Limb characteristics (continued)**	Rutherford classification	2	Captured in perfusion/peripheral arterial disease	Skin temperature	1	
	Trans-Atlantic Inter-Society Consensus Document on Management of Peripheral Arterial Disease (TASC II classification)	3	Captured in perfusion/peripheral arterial disease	Postural instability	1	
	Palpable peripheral pulse	2	Captured in perfusion/peripheral arterial disease	Electromyography	1	
	Peripheral neuropathy	1	Captured in diabetes-related neuropathy			
	Vibration perception threshold	1	Captured in diabetes-related neuropathy			
	Chronic limb-threatening ischaemia (CLTI)	1	Captured in perfusion/peripheral arterial disease			
	Electromyography	1				
	Postural instability	1				
	Saint Elian Wound Score System (SWESS score)	1	Split into individual components			
	Vessel calcification	1				
	Infectious Diseases Society of America (IDSA) classification	1	Split into individual components			
	Diabetic foot ulcer score (DFS)	1	Split into individual components			
	Ulcer Site, Ischemia, Neuropathy, Bacterial infection, Area, and Depth (SINBAD score)	1	Split into individual components			
	Perfusion, extent/size, depth/tissue loss, infection and sensation classification (PEDIS classification)	1	Split into individual components			
	National Pressure Ulcer Advisory Panel and the European Pressure Ulcer Advisory Panel grade (NPUAP/EPUAP grade)	1	Split into individual components			
	Harikrishna peri-wound skin classification	1	Split into individual components			
	Gangrene	1	Captured in perfusion/peripheral arterial disease			

Descriptor domain	Descriptor as identified through systematic review	Number of studies reporting	Comments	Descriptor as taken through to Delphi Survey round 1 after rationalisation by the steering group	Number of studies reporting	Comments
**Limb characteristics (continued)**	Armstrong classification	1	Split into individual components			
	Bates-Jensen score	1	Split into individual components			
	Fontaine staging	1	Captured in perfusion/peripheral arterial disease			
	Limb cold	1	Captured in perfusion/peripheral arterial disease			
	Skin temperature	1	Captured in perfusion/peripheral arterial disease			
	Claudication distance	1	Captured in perfusion/peripheral arterial disease			
**Ongoing medical DFU interventions**	Current use of antibiotics	6		Current use of antibiotics	6	
	Offloading treatments	5		Offloading treatments	5	
	Duration of antibiotic therapy	2		Duration of antibiotic therapy	2	
	Number of dressing changes required	1		Number of dressing changes required	1	
	Duration of healing	1	Difficult to define and capture			
**Previous surgical DFU interventions**	Previous revascularization	4		Previous amputation	15	
	Previous amputation	15		Previous revascularization	4	
**Medication history**	Insulin therapy	23		Insulin therapy	23	
	Hypoglycaemic medications	9		Oral hypoglycaemic medications	9	
	Statins	6		Statins	6	
	Antiplatelets	2		Antiplatelets	4	
	Angiotensin-converting enzyme inhibitor/ Angiotensin II receptor blocker	2	Captured in antihypertensives	Antihypertensives	5	
	Beta-blocker/Calcium channel blocker	2	Captured in antihypertensives	Anticoagulants	1	
	Anticoagulants	1				
	Anti-hypertensives	1				
	Aspirin	2	Captured in antiplatelets			

Descriptor domain	Descriptor as identified through systematic review	Number of studies reporting	Comments	Descriptor as taken through to Delphi Survey round 1 after rationalisation by the steering group	Number of studies reporting	Comments
**Biochemical measurements**	HbA1c	60	Captured in glycaemic control	Glycaemic control	75	New descriptor
	Serum creatinine	20	Captured in renal function	Renal function	30	New descriptor
	Fasting blood glucose	11	Captured in glycaemic control	Lipid profile	19	New descriptor
	Haemoglobin levels	10		Measures of inflammation or infection	18	New descriptor
	Serum albumin	10	Captured in liver function	Liver function	15	New descriptor
	Serum c-reative protein	8	Captured in measures of inflammation or infection	Haemoglobin levels	10	
	Serum cholesterol	7	Captured in lipid profile	Measures of metabolism	7	New descriptor
	Serum leucocytes	7	Captured in measures of inflammation or infection	Measures of coagulation	3	New descriptor
	Serum triglyceride	4	Captured in lipid profile	Magnesium levels	2	
	Serum low density lipoprotein	4	Captured in lipid profile			
	Serum ALT	3	Captured in liver function			
	estimated glomerular filtration rate (eGFR)	3	Capture in renal function			
	Serum high density lipoprotein	3	Captured in lipid profile			
	2-hours post-prandial glucose	2	Captured in glycaemic control			
	Magnesium levels	2				
	Serum Blood Urea Nitrogen	2	Captured in renal function			
	Creatinine clearance	2	Captured in renal function			
	Urinary albumin:creatinine ratio	2	Captured in renal function			
	ESR	2	Captured in measures of inflammation or infection			
	Plasma malondialdehyde concentrations	2	Captured in measures of metabolism			
	Platelets	2	Captured in coagulation			
	Serum vascular endothelial growth factor	1	Captured in measures of metabolism			
	Serum interleukin-6	1	Captured in measures of metabolism			
	Nitric oxide	1	Captured in measures of metabolism			
	Plasma total antioxidant capacity	1	Captured in measures of metabolism			
	Total plasma glutathione	1	Captured in measures of metabolism			
	Fibrinogen	1	Captured in coagulation			
	Serum Tumour Necrosis Factor-alpha	1	Captured in measures of metabolism			
	Serum very low density lipoprotein	1	Captured in lipid profile			
	Serum aspartate aminotransferase (AST)	1	Captured in liver function			
	Serum gamma-glutamyl transferase (GGT)	1	Captured in liver function			

Descriptor domain	Descriptor as identified through systematic review	Number of studies reporting	Comments	Descriptor as taken through to Delphi Survey round 1 after rationalisation by the steering group	Number of studies reporting	Comments
**Biochemical measurements (continued)**	Quantitative insulin sensitivity check index	1	Captured in glycaemic control			
	Homeostatic Model Assessment for Insulin Resistance	1	Captured in glycaemic control			
	Serum urea	1	Captured in renal function			
**Physiological measurements**	Systolic blood pressure	6		Systolic blood pressure	6	
	Heart rate	2		Heart rate	2	
**Quality of life, function and symptoms**	Ambulatory status	8		Ambulatory status	8	
	Activities of Daily Living (ADL)/ Barthel Index	2	Renamed to disease-specific quality of life	The level of independence with the activities of daily living	1	
	Activities of Daily Living (ADL) limitations	1	Renamed to the level of independence with the activities of daily living	Duration of physical activity per day	2	
	Duration of exercise/day	1	Captured in duration of physical activity per day	Assessment of the level of disability caused by neuropathy	1	
	Duration of activity per day	1	Captured in duration of physical activity per day	Emotional burden	1	
	Neuropathy disability score (NBS)	1	Renamed to assessment of the level of disability caused by neuropathy	Health-related quality of life	1	
	Emotional burden	1		Disease-specific quality of life	2	
	Health-related quality of life	1		Assessment of Illness Perception	1	
	Illness Perception Questionnaire	1	Renamed to assessment of illness perception	Living arrangements	1	
	Living arrangements	1		Primary caregiver role	1	
	Primary caregiver role	1				

ESM Table 1. Rationalisation of the descriptor longlist prior to Delphi Round 1.

Descriptors for potential inclusion in CDS	Reason for exclusion
Cause of ulceration	Captured in 'perfusion', 'peripheral artery disease' and 'diabetes-related neuropathy'
Vessel calcification	Assessment of perfusion/presence of PAD is more objective and clinically relevant
Extent of skin undermining	Too subjective
Peri-wound oedema	Too subjective
Description of wound edges	Too subjective
Type of bacterial wound infection	Presence of infection deemed to be more important than the causative microorganism
Ulcer symptoms	Captured in 'foot pain' and 'number of dressing changes required'

ESM Table 2. Critical descriptors excluded after completion of the two rounds of the Delphi survey.

Descriptors without consensus	Reason for exclusion
Electromyography	Too niche for a core descriptor set
Genitourinary disorder	Not directly clinically relevant to DFU
Hepatobiliary disorder	Not directly clinically relevant to DFU
Marital status	Not directly clinically relevant to DFU
Magnesium levels	Not directly clinically relevant to DFU
Neighbourhood income quintile	Not relevant to all healthcare settings
Gastrointestinal disorder	Not directly clinically relevant to DFU
Measures of metabolism	Too niche for a core descriptor set
Income	Not relevant to all healthcare settings
Urban status	Not relevant to all healthcare settings
Arthritis	Not directly clinically relevant to DFU
Employment/profession	Not directly clinically relevant to DFU
Alcohol intake	Not directly clinically relevant to DFU
Family history of diabetes mellitus	Not directly clinically relevant to DFU
Education	Not directly clinically relevant to DFU
Tumours	Not directly clinically relevant to DFU
Liver function	Not directly clinically relevant to DFU
Heart rate	Not directly clinically relevant to DFU
Musculoskeletal disorder	Not directly clinically relevant to DFU
Previous deep venous thrombosis	Not directly clinically relevant to DFU
Dietary habits	Not directly clinically relevant to DFU
Metabolic disorder	Not directly clinically relevant to DFU
Primary caregiver role	Captured within other descriptors related to quality of life
Ophthalmic disorder	Key metric captured in 'diabetes-related retinopathy'
Postural instability	Too subjective
Coagulation	Not directly clinically relevant to DFU
Living arrangements	Not directly clinically relevant to DFU
Lipid profile	Captured in 'hyperlipidaemia'
Systolic blood pressure	Captured in 'hypertension'
Central nervous system disorder	Key metric captured in 'prior stroke'
Emotional burden	Captured within other descriptors related to quality of life
Skin temperature	Captured in 'perfusion', 'peripheral artery disease'
Antihypertensives	Captured indirectly in 'hypertension'
Laterality of the limb involved	Not directly clinically relevant to DFU
Hyperlipidaemia	Pragmatic decision not to include to ensure the final CDS is practicable
Duration of physical activity per day	Captured in 'ambulatory status' and 'the level of independence with the activities of daily living'
Haemoglobin levels	Captured in 'Anaemia'
Anticoagulants	Not directly clinically relevant to DFU
Assessment of the level of disability caused by neuropathy	Captured within other descriptors related to quality of life
Assessment of Illness Perception	Captured within other descriptors related to quality of life

ESM Table 3. Descriptors without consensus excluded after completion of the two rounds of the Delphi survey.
